# Correction: Hsu et al. Hyaluronan: An Architect and Integrator for Cancer and Neural Diseases. *Int. J. Mol. Sci.* 2025, *26*, 5132

**DOI:** 10.3390/ijms262311691

**Published:** 2025-12-03

**Authors:** Che-Yu Hsu, Hieu-Huy Nguyen-Tran, Yu-An Chen, Kuan-Ting Lee, Tzong-Yuan Juang, Ming-Fu Chiang, Shin-Yi Liu, Nan-Shan Chang

**Affiliations:** 1 Department of Medical Laboratory Science and Biotechnology, College of Medicine, National Cheng Kung University, Tainan 70101, Taiwan; bryanhsuks@gmail.com; 2 Graduate Institute of Biomedical Sciences, College of Medicine, China Medical University, Taichung 40402, Taiwan; nguyentranhieuhuy@gmail.com; 3 Graduate Institute of Medical Genomics and Proteomics, College of Medicine, National Taiwan University, Taipei 10617, Taiwan; momofish0716@gmail.com; 4 Graduate Institute of Medicine, College of Medicine, Kaohsiung Medical University, Kaohsiung 80708, Taiwan; ayta860404@gmail.com; 5 Department of Cosmeceutics, China Medical University, Taichung 40402, Taiwan; tyjuang@mail.cmu.edu.tw; 6 Department of Neurosurgery, Fu Jen Catholic University Hospital, Taipei 24352, Taiwan; chiang66@gmail.com; 7 Department of Biomedical Imaging and Radiological Science, China Medical University, Taichung 40402, Taiwan; 8 Department of Neurochemistry, New York State Institute for Basic Research in Developmental Disabilities, New York, NY 10314, USA

After the publication of our review article [1], one of the referenced manuscripts was found to have been retracted. To enhance the quality of the manuscript, we decided to replace the previous reference number 52 [2] in the original manuscript with a new reference [3] at the same position in the reference list.

The original reference number 52 is as follows: Huang, C.; Yoon, C.; Zhou, X.H.; Zhou, Y.C.; Zhou, W.W.; Liu, H.; Yang, X.; Lu, J.; Lee, S.Y.; Huang, K. ERK1/2-Nanog signaling pathway enhances CD44(+) cancer stem-like cell phenotypes and epithelial-to-mesenchymal transition in head and neck squamous cell carcinomas. *Cell Death Dis.* **2020**, *11*, 266.

The new replacement for reference 52 is as follows: Prince, M.E.; Sivanandan, R.; Kaczorowski, A.; Wolf, G.T.; Kaplan, M.J.; Dalerba, P.; Weissman, I.L.; Clarke, M.F.; Ailles, L.E. Identification of a subpopulation of cells with cancer stem cell properties in head and neck squamous cell carcinoma. *Proc. Natl. Acad. Sci. USA* **2007**, *104*, 973–978.

This replacement does not affect the meaning of the original text in the published paper. There was no need to adjust the number for each cited reference in the References section. The authors state that the scientific conclusions are unaffected. This correction was approved by the Academic Editor. The original publication has also been updated.

## References

[1] Hsu C.-Y., Nguyen-Tran H.-H., Chen Y.-A., Lee K.-T., Juang T.-Y., Chiang M.-F., Liu S.-Y., Chang N.-S. (2025). Hyaluronan: An Architect and Integrator for Cancer and Neural Diseases. Int. J. Mol. Sci..

[2] Huang C., Yoon C., Zhou X.H., Zhou Y.C., Zhou W.W., Liu H., Yang X., Lu J., Lee S.Y., Huang K. (2020). ERK1/2-Nanog signaling pathway enhances CD44(+) cancer stem-like cell phenotypes and epithelial-to-mesenchymal transition in head and neck squamous cell carcinomas. Cell Death Dis..

[3] Prince M.E., Sivanandan R., Kaczorowski A., Wolf G.T., Kaplan M.J., Dalerba P., Weissman I.L., Clarke M.F., Ailles L.E. (2007). Identification of a subpopulation of cells with cancer stem cell properties in head and neck squamous cell carcinoma. Proc. Natl. Acad. Sci. USA.

